# High Content Analysis of Human Pluripotent Stem Cell Derived Hepatocytes Reveals Drug Induced Steatosis and Phospholipidosis

**DOI:** 10.1155/2016/2475631

**Published:** 2016-01-05

**Authors:** Arvind Pradip, Daniella Steel, Susanna Jacobsson, Gustav Holmgren, Magnus Ingelman-Sundberg, Peter Sartipy, Petter Björquist, Inger Johansson, Josefina Edsbagge

**Affiliations:** ^1^Takara Bio Europe AB (Former Cellectis AB/Cellartis AB), Arvid Wallgrens Backe 20, 413 46 Göteborg, Sweden; ^2^Novo Nordisk A/S, Stem Cell Development, 2880 Bagsværd, Denmark; ^3^Horizon Discovery Ltd, 7100 Cambridge Research Park, Waterbeach, Cambridge CB25 9TL, UK; ^4^Systems Biology Research Center, School of Bioscience, University of Skövde, 541 28 Skövde, Sweden; ^5^Department of Physiology and Pharmacology, Section of Pharmacogenetics, Karolinska Institutet, 171 77 Stockholm, Sweden; ^6^AstraZeneca R&D, GMD CVMD GMed, 431 83 Mölndal, Sweden; ^7^NovaHep AB, Arvid Wallgrens Backe 20, 413 46 Göteborg, Sweden

## Abstract

Hepatotoxicity is one of the most cited reasons for withdrawal of approved drugs from the market. The use of nonclinically relevant* in vitro* and* in vivo* testing systems contributes to the high attrition rates. Recent advances in differentiating human induced pluripotent stem cells (hiPSCs) into pure cultures of hepatocyte-like cells expressing functional drug metabolizing enzymes open up possibilities for novel, more relevant human cell based toxicity models. The present study aimed to investigate the use of hiPSC derived hepatocytes for conducting mechanistic toxicity testing by image based high content analysis (HCA). The hiPSC derived hepatocytes were exposed to drugs known to cause hepatotoxicity through steatosis and phospholipidosis, measuring several endpoints representing different mechanisms involved in drug induced hepatotoxicity. The hiPSC derived hepatocytes were benchmarked to the HepG2 cell line and generated robust HCA data with low imprecision between plates and batches. The different parameters measured were detected at subcytotoxic concentrations and the order of which the compounds were categorized (as severe, moderate, mild, or nontoxic) based on the degree of injury at isomolar concentration corresponded to previously published data. Taken together, the present study shows how hiPSC derived hepatocytes can be used as a platform for screening drug induced hepatotoxicity by HCA.

## 1. Introduction

The liver is the most important and susceptible organ in drug toxicity being functionally interposed between site of absorption and systemic circulation [1]. Drug induced liver injury (DILI) is broadly classified into intrinsic (dose dependent and usually predictable) and idiosyncratic (does not depend on dose and unpredictable). DILI has been reported as the major reason for withdrawal of approved drugs from the market [2]. Nearly 90% of the lead candidates identified by current* in vitro* screens fail to become drugs and about 50–60% of drugs progressing to clinical trials fail in the late stages of drug development [3, 4]. This raises a need for devising more relevant and effective screening strategies for identifying new candidate drugs (CDs), with low risk to cause DILI [5]. DILI in particular makes it more difficult owing to several mechanisms of toxicity being involved. In addition, complex interactions with the immune system, exposure to viral infections, and genetic background of individuals affect the sensitivity of DILI [6]. Different compounds have their own sequential pattern through which they manifest an injury. Also a single drug can have multiple effects with several mechanisms of toxicity [1]. Despite the numerous animal and* in vitro* models available, currently used assays have low concordance with human liver toxicity [7, 8]. The cells need to be of human origin with functional drug metabolic competence due to substantial species differences.

High content analysis (HCA) is a powerful cell based screening method showing high sensitivity and specificity in combination with an appropriate cell source. This technology employs simultaneous measurement of multiple endpoints which are relevant to the mechanisms involved in toxicity [9]. Several cellular models are being used to study drug metabolism and toxicity. Some of the more well-established models are primary cell cultures, immortalized cell lines, intracellular fractions, precision cut liver slices, and whole perfused livers [10]. However, due to different limitations of these models, there is currently no ideal* in vitro* assay for testing hepatotoxicity.

**Figure 1:**
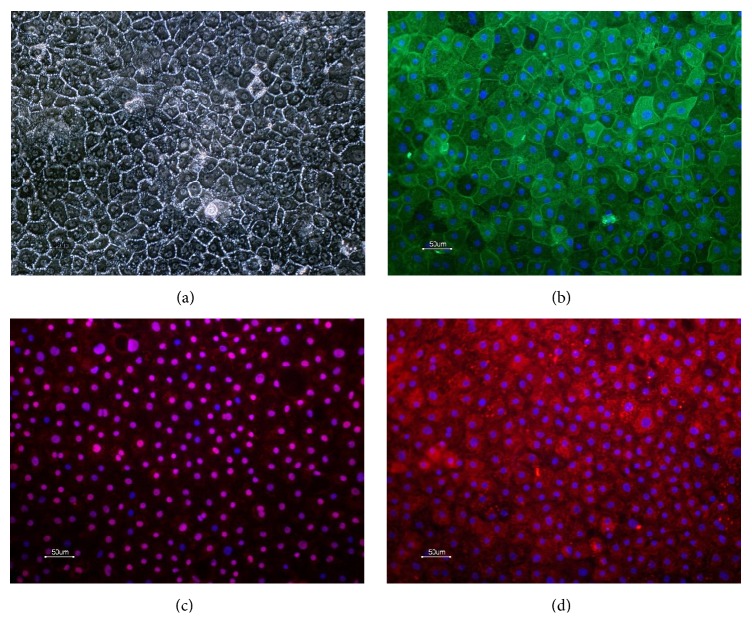
Morphology of hiPSC derived hepatocytes (a) and expression of hepatic markers cytokeratin 18 (b), HNF4*α* (c), and alpha-1-antitrypsin (d). Scale bars equal 50 *μ*m.

Human pluripotent stem cells (hPSC) possess two very important capabilities, infinite self-renewal and the ability to differentiate into any cell type in the human body. Hence, they are being explored as a promising source of functional human hepatocytes. Hepatocytes can be derived from both human* embryonic* stem cells (hESCs) and human* induced pluripotent* stem cells (hiPSCs) [11, 12]. These cells have several significant advantages over existing systems, such as the fact that they are of human origin, the fact that they allow cell manufacturing with consistency between batches with an endless supply of cell material, and the opportunity to select genetic background of the starting material [13, 14]. Recently, advances in differentiating hESC and hiPSC to hepatocytes have been made generating highly pure cultures of hiPS derived hepatocyte like cells expressing hepatic markers and functional drug metabolizing cytochrome P450 enzymes. The hiPSC derived hepatocytes used in this study were differentiated from Cellartis human iPSC line, ChiPS4, using Cellartis DE Diff Kit and Hepatocyte Diff Kits (referred to as hiPS-HEP in previous publications) [15, 16]. The hiPS-hepatocytes exhibit typical hepatic morphology, expressing many hepatic markers (Figure 1), and are capable of metabolizing drugs via the cytochrome P450 (CYP) families 1A and 3A [15].

In the present study, we show that the hiPSC derived hepatocyte can serve as a platform for monitoring drug induced steatosis and phospholipidosis by HCA following mechanistic endpoints such as viability, nuclear changes, mitochondrial membrane potential (MMP), reactive oxygen species (ROS), and plasma membrane permeability (PMP). The hiPS-hepatocyte platform was benchmarked against the well-established HepG2 cells. Based on the toxic mechanisms involved, the chemicals amiodarone, doxycycline, tetracycline, and sodium citrate were categorized as severe, moderate, mild, and nontoxic. The assays for various parameters were robust and reproducible between wells, plates, and batches and thus hiPSC derived hepatocytes are a promising* in vitro* cell system for toxicity assessment by HCA.

## 2. Materials and Methods 

### 2.1. Human Induced Pluripotent Stem Cells and Hepatic Differentiation

The hiPSC line ChiPSC4 (Takara Bio Europe AB) was derived as described before using human dermal fibroblasts [15]. ChiPSC4 was maintained and cultured under standard conditions in the Cellartis DEF-CS with continuous passaging twice a week according to manufacturer manual (Takara Bio Europe AB).

For hepatic differentiation, a serum- and feeder-free procedure recapitulating liver development was applied. First, ChiPSC4 was guided to differentiate into definitive endoderm using the Cellartis DE Diff Kit (Takara Bio Europe AB; Y30030), containing complete media and coating solution for differentiation of hPSC to DE cells in 2D culture. On day 7 of differentiation, the DE cells were enzymatically dissociated and reseeded into fibronectin coated 96-well plates. Briefly, fibronectin solution was prepared by diluting fibronectin (Sigma; F0895) 1 : 20 to 50 *μ*g/mL in D-PBS^+/+^ (Life Tech; 14200-067). Wells of 96-well plates were coated by adding fibronectin solution 0.15 mL per cm^2^ to the wells and let to incubate for >60 min at RT. DE cells were enzymatically detached using TrypLE Select (Life Tech; 12563-011) 0.1 mL/cm^2^ and incubated for 3–5 min at 37°C. The enzymatic reaction was stopped by adding 10% KO-SR (Life Tech; 10828-028) in D-PBS^−/−^ to achieve a 1 : 1 dilution of the cell suspension. Next, the cell suspension was centrifuged for 5 min at 300 g at RT, the supernatant was removed, and the cell pellet was resuspended in warm Hepatocyte Thawing and Seeding medium (Cellartis Hepatocyte Diff Kit; Takara Bio Europe AB; Y30050). Prior to seeding of cells, excess coating was removed and 150 K DE cells/cm^2^ were seeded in the 96-well plates. On days 9 and 11 (counted from the start of ChiPSC4 differentiation), medium was changed using Hepatocyte Progenitor Medium (Cellartis Hepatocyte Diff Kit). On day 14 and onwards, medium changes were performed every second or third days using warm Williams Medium E (Life Tech; 32551-087) supplemented with 0.1% PEST, HCM Single Quots (Lonza; CC-4182; GA-1000 was omitted), 10 ng/mL Oncostatin M (PromoKine; C-65020), 40 ng/mL Hepatocyte Growth Factor (PromoKine; C-64530), 0.1 *μ*M dexamethasone (Sigma; D8893), and 1.4 *μ*M BIO (Sigma; B1686).

The differentiation procedure described above corresponds to Cellartis hiPS-HEP by Takara Bio Europe AB (Göteborg, Sweden).

### 2.2. Immunocytochemistry

Human iPSC derived hepatocytes were stained as previously described in Ulvestad et al. [15]. The primary antibodies used in this study were rabbit anti-*α*1-antitrypsin (1 : 200, A0012, DAKO), mouse anti-CK18 (1 : 100, M7010, DAKO), and rabbit anti-HNF4*α* (1 : 300, sc-8987, SantaCruz Biotechnology). The following secondary antibodies were used and purchased from Life Technologies: donkey anti-rabbit Alexa Fluor 488 IgG (1 : 1000, A21206), donkey anti-rabbit Alexa Fluor 594 IgG (1 : 1000, A21207), and goat-anti-mouse Alexa Fluor 488 (1 : 500, A11029).

### 2.3. Materials for HCA

The fluorescent dyes (Hoechst 33342, HCS LipidTOX Green neutral lipids (H34475), HCS LipidTOX Red phospholipidosis (H34351), MitoTracker orange CMTMRos (M7510), carboxy-H2DCFDA (C400), and TOTO-3 Iodide (T3604)) were from Life Technologies, Invitrogen. The CellBIND 96 Well Flat Clear Bottom Black Polystyrene Microplates were purchased from Corning.

### 2.4. Selection of Compounds

Three compounds (amiodarone hydrochloride, doxycycline, and tetracycline hydrochloride) known to induce hepatotoxicity through steatosis and phospholipidosis were studied (Table 1) [17]. As a negative control, sodium citrate, a nontoxic agent, was included. Positive controls were included to assess the quality of testing in each plate. Compounds with known responses were added in triplicate for each endpoint being measured. The following drugs/agents were used as positive controls for different toxic read-outs: Cyclosporin A (30 *μ*M) for neutral lipids, propranolol (30 *μ*M) for phospholipids, mitochondrial uncoupler FCCP (100 *μ*M) for mitochondrial membrane potential, and tert-Butyl hydroperoxide (TBHP) luperox (100 *μ*M) for reactive oxygen species. When the control treated values deviated more than three standard deviations away from the mean of the other two wells, they were considered as outliers. If more than three outliers occurred in a plate, the experiment was discarded and repeated.

### 2.5. HepG2 Cell Culture

HepG2 cells (HB-8065, ATCC) were cultured according to the provider's instructions and as previously described [15]. Briefly, the cells were grown in DMEM supplemented with 10% heat inactivated FBS, 1% penicillin-streptomycin (PEST), 1% sodium pyruvate, and 1% nonessential amino acids. Cells were passaged at 1 : 8 or 1 : 6 every 3-4 days when they reach 75–80% confluence. Seeding density was optimized for HCA application to ensure that the cells were in a monolayer (5000 cells per well in 96-well plates) for precise imaging. The cells were grown for 48 h to attach and stabilize. The experiments performed with HepG2 cells were designed to ensure that each assay was performed with cells in a similar growth phase.

### 2.6. Assay Procedure

**Figure 2:**
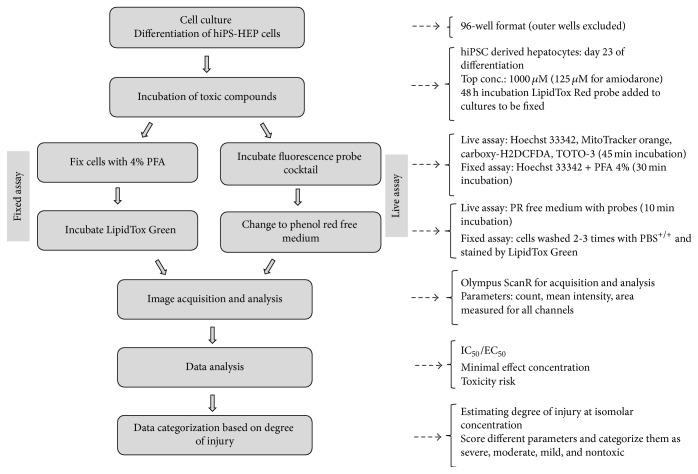
HCA assay scheme for screening drug induced steatosis and phospholipidosis.

The cells were treated for 48 h with compounds at varying concentrations (half log dilution from the top concentration). The highest concentrations were fixed approximately 100-fold their C_max⁡_, 1000 *μ*M for doxycycline, tetracycline, and sodium citrate, and 125 *μ*M for amiodarone. The stock solutions were prepared in DMSO or water and were diluted in culture medium to obtain the final concentration. Each concentration was assayed in triplicate wells. Vehicle control wells were included and the final DMSO concentration in the medium never exceeded 0.5% (v/v). Each batch had two plates each for fixed and live assays. The experimental process is illustrated in Figure 2.

### 2.7. Probes

Hoechst 33342, a cell-permeant nuclear dye, was used for measuring nuclei changes and cell number. LipidTOX Green neutral lipids and LipidTox Red phospholipidosis were used for identifying neutral lipid and phospholipid formation within the cells. MMP changes (Δ*ψ*m) within the cell were measured using MitoTracker orange. Carboxy-H2DCFDA (6-carboxy-2′,7′-dichlorodihydrofluorescein diacetate) an acetylated fluorescent was used for measuring ROS. And finally, TOTO-3 was used for assessing PMP.

The fluorescence probes were grouped into two sets according to their optical compatibility and requirement of fixed or live material. The probe concentrations were optimized and were grouped in such a way that their absorption and emission spectrum did not overlap to avoid spectral bleed-through while taking the spectral range of quadruple filter being used into consideration. The fluorescent probes for neutral lipids and phospholipids required the cells to be fixed and were assigned to the fixed assay. The probes MitoTracker orange, carboxy-H2DCFDA, and TOTO-3 were assigned to the live assay. Hoechst 33342 was included in both the fixed and live assays.

### 2.8. Administration of Probes

Following compound incubation, probes were administered to the cultures of the live assay. 50 *μ*L medium containing MitoTracker orange, carboxy-H2DCFDA, TOTO-3, and Hoechst probes was loaded to the cells with 100 *μ*L medium to a final concentration of 300 nM, 25 *μ*M, 1 *μ*M, and 1X, respectively. After incubation for 45 min at optimal cell culture conditions, the cells were changed to phenol red (PR) free medium for live cell imaging. For fixed assay, LipidTox Red probe was added with the compounds, and after 48 h the cells were fixed in 4% PFA supplemented with 1X Hoechst stain for 30 min at room temperature. Subsequently, cells were washed with PBS^+/+^ and 1X LipidTOX Green stain diluted in PBS^+/+^ was incubated with the cells for 30 min at room temperature. The plates were imaged without removing the buffer with probe.

### 2.9. Image Acquisition

The cells were imaged using an Olympus ScanR system. Prior to image acquisition, the heating system and CO_2_ gas flow were started to ensure optimal conditions in the ScanR chamber for live cell analysis. The incubator was set to 37°C with 5% CO_2_ and relative humidity of 90%. The LUCPLFLN 20X long distance objective with NA 0.45 was used to image distinct fluorescence channels. In order to cover the entire well and also to capture maximum cell events sixteen fields per well (4 × 4 tile grid spread equally) were imaged. The channel exposure time for LipidTOX Green, LipidTOX Red, MitoTracker orange, and carboxy-H2DCFHDA were set based on the fluorescence of the negative control wells and were set constant within each plate. Exposure time for Hoechst and TOTO-3 was set in the range of 1–10 ms depending on cellular fluorescence intensity.

### 2.10. Image Analysis

The acquired images were analyzed using ScanR analysis program from Olympus. Background correction was applied for all images with a constant filter size value before quantification. During analysis, out-of-focus images were discarded to avoid deluding intensity values. Hoechst fluorescence signal identifying nuclei was used as the main object for cell count and other parameters measuring nuclear changes. To define main object, intensity threshold was used as object finding module. Watershed algorithm was applied to separate clusters along the indentations along the contours of the clusters to get individual cells. All other fluorescence signals were considered as subobjects. A ring area was applied around the main objects/nuclei with a set distance marking cytosolic region around the nucleus. This area is assigned* mask* and all other parameters besides the nucleus (the main object)-related parameters were measured within the mask area. Selected parameters such as count, total intensity, mean intensity, and area were estimated for each fluorescence channel. LipidTox Green and LipidTOX Red fluorescence defined for neutral lipids and phospholipids, respectively, were measured as spots for estimating number of lipid/phospholipid droplets per cell. MMP, ROS, and PMP parameters were estimated using intensity module. The analyzed parameters were exported to Microsoft excel for further calculations. The values for each parameter were normalized to the vehicle control and are presented as percentage of vehicle control.

### 2.11. Data Analysis

For each parameter analyzed, the dose response curves were plotted and IC_50_/EC_50_ values were generated when possible with GraphPad Prism 5 using nonlinear four-parameter logistic curve fit (least squares). The minimal effect concentration (MEC) was defined as the lowest concentration that produced significant difference (*p* ≤ 0.05) when compared to the vehicle control. The toxicity risk (TR) for each compound was defined as ratio of minimal effective concentration to the maximum plasma concentration of the drug (C_max⁡_). Steatotic risk index (SRI) was calculated as the ratio of the MEC for neutral lipid accumulation or for ROS generation to C_max⁡_.

In order to classify compounds according to their toxicity potential, the level of change was calculated for each parameter at isomolar concentration of 100 *μ*M (except for amiodarone HCl at 125 *μ*M). The scoring sheet used for estimating the degree of injury was adapted from Tolosa et al. [18]. Four different scores were assigned according to the level of variation when compared to the vehicle control; 0 (variation lower than 20%), 1 (variation ±20–40%), 2 (variation ±40–60%), and 3 (variation ±60–100%). The percentage change for steatosis and phospholipidosis was in a different range, so different levels were established. The scores were assigned as 0 (variation lower than 25%), 1 (variation between 25 and 150%), 2 (variation between 150 and 300%), and 3 (variation >300%). The individual scores for each parameter were summed up to estimate the severity or the degree of injury of the compound. The compounds were classified based on the degree of injury as severely toxic (≥15), moderately toxic (6–15), mildly toxic (1–5), and nontoxic (0). The order in which the compounds were classified was compared between hiPS-HEP and HepG2 cells.

### 2.12. Assessment of Predictivity

Sensitivity was measured as the proportion of toxic drugs testing positive, TP/(TP + FN), where TP is the number of toxic compounds testing positive and FN is the number of toxic drugs testing negative. Specificity was measured as proportion of nontoxic drugs testing negative, TN/(TN + FN), where TN is the number of nontoxic drugs testing negative and FN is the number of nontoxic drugs testing positive. For predicting overall toxicity producing a positive response, a compound should have a clear dose response relationship and the magnitude of effect had to be biologically relevant. The compound was not considered positive if the effects were seen only at the highest concentration unless subsequent effect was measured for either of the parameters at lower concentration. An effect was considered positive when the parameter analyzed showed significant difference of *p* ≤ 0.05 when compared to the control.

### 2.13. Assay Imprecision

To estimate the degree of random variation and artifacts in the assay, imprecision in different parameters was determined. The variations were estimated between the following: (1) well-to-well within a plate, (2) plate-to-plate within a batch, and (3) batch-to-batch for every parameter measured. Negative control wells were used to assess imprecision in cell count, nuclear changes, and plasma membrane permeability. Positive control wells were used for assessing imprecision in MMP, steatosis, phospholipidosis, and ROS. Values were considered as outliers and excluded, when they were more than three standard deviations away from the corresponding mean values for each parameter.

To estimate well-to-well coefficient of variance, mean, standard deviation, and CV% were calculated for every control well (*N* = 3) and an average CV% of all the eight plates was reported for well-to-well variation. The well-to-well mean values for each parameter within a plate (*N* = 2) were then averaged and its CV% was calculated for plate-to-plate variation. For batch-to-batch variance, the mean values from each plate were averaged and its CV% was reported (*N* = 4).

### 2.14. Statistical Analysis

Test compounds and controls were measured in triplicate with at least three independent experiments for both hiPS-HEP and HepG2 cells. The statistical analysis was performed using one-way ANOVA, with Dunnett's test as the post hoc method. A *p* value equal to or below 0.05 was considered statistically significant.

## 3. Results 

### 3.1. Characterization of Human iPS Derived Hepatocytes

The morphological characteristics of hiPSC derived hepatocytes were monitored at the start of the experiment and during toxicity dosing. The hiPSC derived hepatocytes displayed distinct morphology closely resembling human primary hepatocytes* in vitro *(Figure 1). They formed a monolayer of flat polygonal shaped cells and were often binucleated. The hiPSC derived hepatocytes expressed typical hepatic markers, for example, cytokeratin 18, HNF4*α*, and alpha-1-antitrypsin, uniformly in the cultures (Figure 1). It was previously described and shown that the hiPS derived hepatocytes (denoted Cellartis hiPS-HEP) express functional cytochrome P450 activity of CYP1A and 3A [15].

### 3.2. HCA Assessment of Drug Induced Steatosis and Phospholipidosis

Multiplexing different probes gave an overview of the mechanisms being affected by a compound. Different parameters were measured within a specific cytoplasmic mask around single- or binuclei of the cells. Dose response curves of parameters measured for amiodarone, doxycycline, tetracycline, and sodium citrate in hiPSC derived hepatocytes and HepG2 cultures were quite consistent between the two cell systems. However, the sequence of parametric changes varied between compounds and the two cell sources.

**Figure 3:**
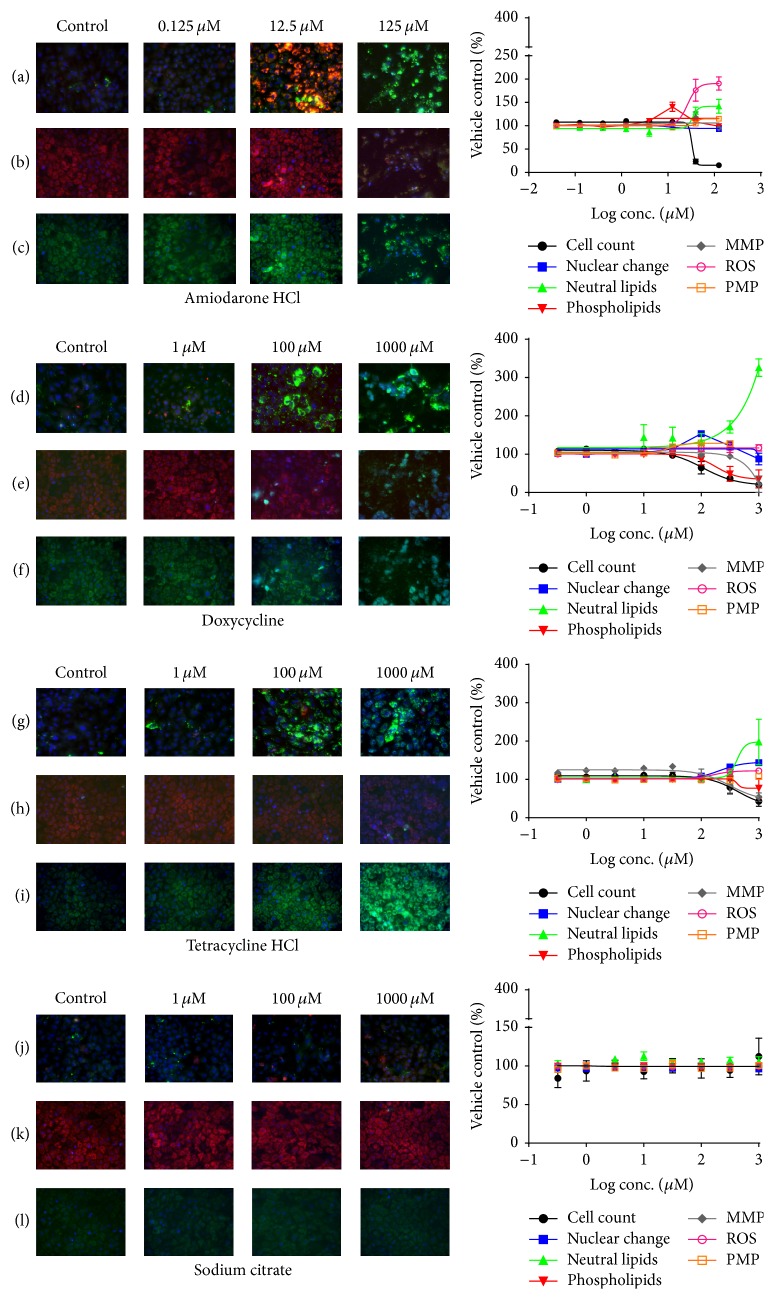
Representative images of dose response effects of amiodarone HCl (a–c), doxycycline (d–f), tetracycline hydrochloride (g–i), and sodium citrate (j–l) at three different concentrations in hiPSC derived hepatocytes. Nuclei were detected by Hoechst 33342 (blue) staining in all images. Fluorescence of LipidTOX neutral lipids (green) and LipidTOX phospholipids (red) in rows (a), (d), (g), and (j) and MMP (red) and PMP (green) in (b), (e), (h), and (k). ROS (green) in (c), (f), (i), and (l), respectively. Values denoted are mean ± SEM (*N* = 3).

**Figure 4:**
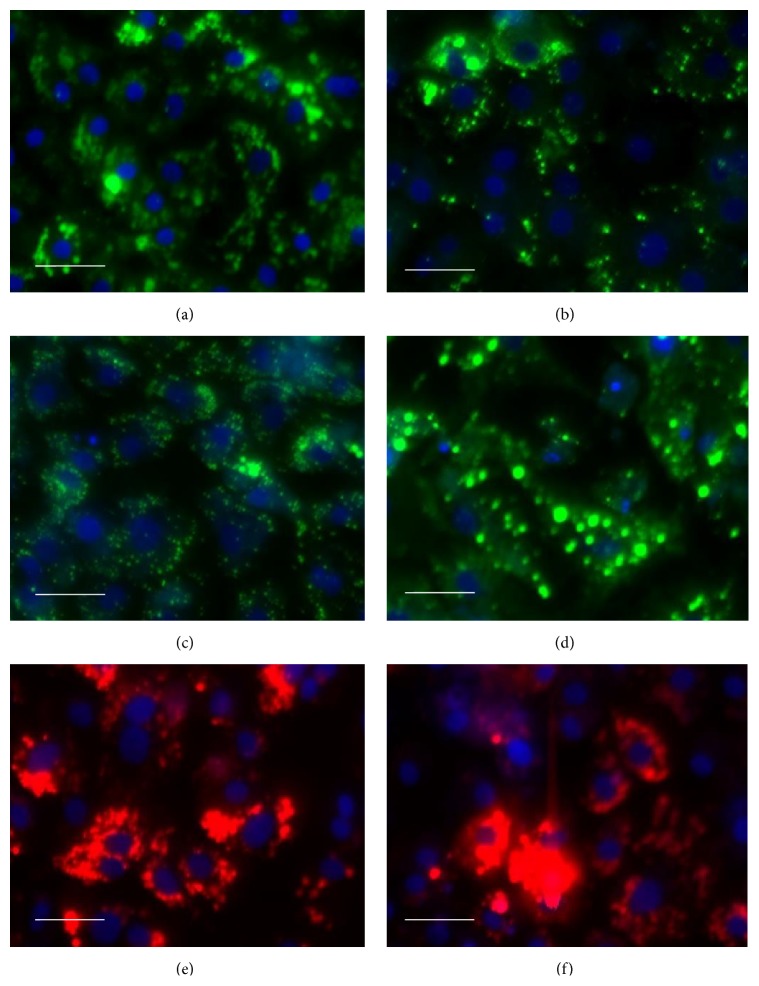
High magnification images of drug induced neutral lipid accumulation in droplets of hiPS derived hepatocytes (LipidTox Green) by amiodarone 12,5 *μ*M (a), doxycycline 32 *μ*M (b), tetracycline 100 *μ*M (c), and cyclosporine A 30 *μ*M (positive control, d) and drug induced phospholipidosis (LipidTox Red) by amiodarone 12,5 *μ*M (e) and propranolol 30 *μ*M (positive control, f). Nuclei are stained blue by DAPI. Scale bar 50 *μ*M.

The dose response effects of amiodarone, doxycycline, tetracycline hydrochloride, and sodium citrate in hiPSC derived hepatocytes are shown in Figure 3. Amiodarone induced both steatosis and phospholipidosis and displayed no cytotoxic effects below 12.5 *μ*M, while the cell count decreased by 76% at 39.5 *μ*M. Sequential events were observed as follows: phospholipid accumulation started at 4 *μ*M, followed by an increase in ROS and subsequent increase in MMP. Hyperpolarization of mitochondrial membrane was detected in doses up to 40 *μ*M where at higher doses the mitochondrial membrane potential dropped. Phospholipids were not detected at concentrations higher than 40 *μ*M. Pronounced steatogenic effects were apparent with 18-fold increase in number of lipid droplets compared to control and in a dose dependent increase of intensity and mean lipid area.  Figure 4 shows high magnification images of lipid droplet formation in hiPSC derived hepatocytes induced by the steatogenic drugs amiodarone, doxycycline, tetracycline, and cyclosporine A and phospholipid accumulation induced by amiodarone and propranolol.

Doxycycline had effects on plasma membrane permeability (PMP) at low concentrations starting from 32 *μ*M. The cell count was reduced by 50% at 100 *μ*M and significant increase in lipid accumulation was observed at 320 *μ*M. In parallel to lipid accumulation, doxycycline induced a concentration dependent depolarization of the mitochondrial membrane, while ROS levels were unaffected. As expected, no changes were detected for phospholipid parameters.

Tetracycline hydrochloride had minimal cytotoxic effects. The mean nuclear intensity and area were similar to the control up to 100 *μ*M, and at higher concentrations small increases by 12% and 14%, respectively, were observed. The sequence of effects was as follows: at 32 *μ*M the mitochondrial membrane potential (MMP) and the accumulation of lipid droplets increased, at 100 *μ*M the oxidative stress increased, and at 320 *μ*M the nuclear changes were evident for both nuclear intensity and area. Finally, at the highest concentration, PMP was slightly affected by an increase of 8% compared to the control. Accumulation of phospholipids was not detected.

No significant effects were caused by the negative control substance sodium citrate. The parameters measured were consistent over the whole dose range.

### 3.3. Determination of IC_50_/EC_50_

The IC_50_ and EC_50_ values were calculated for each parameter which had a complete range of dose response (Table 2). For dose response curves which had a wide confidence interval the values were treated as ambiguous, thus a direct corelation or comparison between hiPSC derived hepatocytes and HepG2 cell lines could not be established. The values were highly correlated with the concentration at which evident effects were seen. The IC_50_ and EC_50_ values in relation to the parameters investigated aid at determining the likely mechanisms through which the compounds cause toxicity.

From Table 1, it can overall be inferred that the most sensitive parameters were ROS and nuclear area for both the hiPSC derived hepatocytes and HepG2 cells. In general, hiPSC derived hepatocytes had a more complete dose response than HepG2 for the concentrations measured. The most sensitive parameter for amiodarone was nuclear area followed by phospholipid area in hiPSC derived hepatocytes and ROS and cell count for HepG2.

### 3.4. Classifying Compounds by Their Mechanism of Action Using MEC

The minimal effective concentration (MEC) was calculated as the lowest concentration with a significant change (*p* < 0.05) when compared to the control for all parameters (Table 3). The mechanism affected at the lowest concentration was considered as the main mechanism for toxicity (denoted in bold). The significance of cytotoxic signals was estimated by calculating TR and SRI as mentioned under methods [7].

The ranking of sensitivities of the different parameters was different when assessed based on MEC in comparison to IC_50_ or EC_50_ values. This difference likely reflects that the point of first clear significant effect (measured as MEC) measures a low dose enhancement while to reach half maximal mark (which is measured as IC_50_/EC_50_) was prolonged for some parameters.

### 3.5. Predictivity of the Assay: Specificity and Sensitivity

The specificity of the assay in hiPSC derived hepatocytes and HepG2 cells for the three compounds tested was 100%. The number of compounds screened to present a tangible predictivity value is too low in this study to be able to fully address specificity of the two* in vitro* systems. Overall the sensitivity was 100% for nuclear changes, ROS, and steatosis in both hiPSC derived hepatocytes and HepG2. Human iPSC derived hepatocytes appear less sensitive to PMP since one of the three compounds (tetracycline hydrochloride) did not generate any significant changes. However, it is uncertain whether a cytotoxic effect is expected with tetracycline hydrochloride for the concentrations measured. Notably, HepG2 cells that previously have been documented for detecting phospholipidosis were less sensitive than hiPSC derived hepatocytes and failed to detect phospholipid accumulation by amiodarone.

### 3.6. Categorizing Compounds Based on Their Degree of Injury

The degree of injury was estimated at isomolar concentration of 125 *μ*M for amiodarone and 100 *μ*M for doxycycline, tetracycline, and sodium citrate. The scores were assigned for each parameter according to the level of variation in comparison to the control value. The compounds were categorized based on the total scores according to Tolosa et al. [18] and as described in methods (Table 4). Amiodarone and tetracycline were categorized as severely and mildly toxic, respectively, in both cell types. Doxycycline which was moderately toxic in hiPSC derived hepatocytes was categorized as mildly toxic in HepG2. Sodium citrate, a nontoxicant, was mildly toxic in HepG2 and nontoxic in hiPSC derived hepatocytes.

### 3.7. Assay Imprecision

The variance measurement between well-to-well, plate-to-plate, and batch-to-batch variation for each parameter in hiPSC derived hepatocytes and HepG2 is shown in Table 5. Overall, well-to-well variations within a plate had the most precise measurements for both hiPSC derived hepatocytes and HepG2. The variation of cell count between plates and batches, respectively, was high compared to other parameters measured. The higher variances for cell count in live assays could be due to the conditions maintained during imaging.

When comparing the cell sources for preciseness, hiPSC derived hepatocytes were clearly more consistent for live cell analysis. Key parameters like MMP, ROS, PMP, neutral lipid area, nuclear area, and nuclear mean intensity between plates and batches are more precise in hiPSC derived hepatocytes than HepG2 cells. The average variations (CV% ± SD) for the selected parameters were 5.3 ± 3 for hiPSC derived hepatocytes compared to 13.6 ± 7.3 for HepG2. However, intensity values for neutral lipids and phospholipids in fixed assay displayed lower variance in HepG2 cells.

## 4. Discussion

In the present study, we show that hiPSC derived hepatocytes might be highly sensitive and specific as an* in vitro* model for detecting drug induced steatosis and phospholipidosis, as here monitored by HCA for three different compounds. Three test drugs known to induce steatosis and/or phospholipidosis were applied to the hiPSC derived hepatocytes based model system and to a well-established model system, HepG2 cells, for benchmarking. The aim was to investigate the potential of hiPSC derived hepatocytes to serve as a platform for mechanism-based toxicity testing by HCA and its ability to correlate to known clinical toxicity patterns in humans. Since the selected drugs are known to affect cell-health at varying concentrations and through multiple mechanisms, the assay was multiplexed with different fluorescent probes to detect several endpoints.

DILI or drug induced hepatotoxicity is known to have an intrinsic and idiosyncratic mechanism of which the most common effects are mitochondrial impairment, oxidative stress, steatosis, cholestasis, phospholipidosis, and immune mediated and apoptotic or necrotic cell death [1, 17]. Most studies on drug induced steatosis are from clinical studies in humans and as a consequence costly failures of candidate drugs due to toxicity are discovered late in the drug discovery process [8, 19]. There is therefore an urgent need for more relevant and predictive cell based model systems to screen for compounds inducing steatosis and phospholipidosis at an early stage in the drug discovery process.

Phospholipidosis, characterized by excessive intracellular accumulation of phospholipids in lysosomes and subsequent formation of laminar bodies, is multifactorial through several mechanisms [20]. While the biochemical conditions have been well characterized, it is unclear whether drug induced phospholipidosis per se is detrimental to humans. However, many researchers consider it as an indicator for the accumulation of drugs and their metabolites accumulating within the cell, which can have severe implications during chronic exposure [21]. Drug induced liver steatosis has been reported to be caused by multiple mechanism, for example, direct inhibition of *β*-oxidation, impairment of mitochondrial respiratory chain (MRC) giving rise to enhanced ROS formation, mitochondrial dysfunction, and increased triglyceride (TG) synthesis [19, 21]. These different mechanisms are highly related and consequently an early alteration in homeostasis of one or more mechanisms leads to subsequent detrimental effects in the hepatocytes which account for the histopathological findings in drug induced steatosis and phospholipidosis [18, 22, 23]. Compounds are known to undergo repeated oxidation or reduction cycles which produce free radicals exceeding antioxidative threshold, thereby imposing oxidative stress [24]. A subsequent increase in ROS can damage proteins, lipids, or DNA which would in turn cause altered Ca^2+^ homeostasis, lipid peroxidation, or mitochondrial dysfunction [19, 25]. Altered Ca^2+^ can disrupt membrane permeability, influence mitochondrial respiratory chain [26], and also activate proteases and endonucleases leading to necrosis or apoptosis [27]. Alternatively, compounds which directly cause an imbalance in MMP would induce ROS formation causing subsequent cell death [27]. This current study demonstrates that hiPSC derived hepatocytes exposed to well-known drugs reflect the sequential mechanistic effects reported in the literature (Table 1 and Figure 3).

Cell viability in response to 48 h amiodarone treatment has previously been reported for hiPSC derived hepatocytes (hiPS-HEP) and HepG2 in Holmgren et al. 2014 [16] with slightly different outcome compared to what was shown in the current data set. The two cell types showed similar dose response curves for viability measured by the proliferation and viability assay EZ4U in Holmgren et al. study [16], while HCA measurement of viability by cell count revealed that HepG2 cells were more sensitive to amiodarone than the hiPSC derived hepatocytes (Tables 2 and 3). Data suggests that cell count by HCA is a more sensitive method to measure viability.

In the present study, several insights were gained by estimating the IC_50_/EC_50_ values for each parameter measured (Table 2) (excluding values higher than cell counts IC_50_ value, i.e., effects seen after 50% loss of cells). Notably, HepG2 cells depict a poorer predictivity for the different mechanistic parameters at subcytotoxic concentrations compared to hiPSC derived hepatocytes. Most of the parameters measured for amiodarone and doxycycline had IC_50_/EC_50_ values higher than IC_50_ value for cell count in HepG2 cells.

Since IC_50_/EC_50_ for a few parameters had ambiguous values due to the wide dose range tested, MEC, a system created for estimating the toxicity potential to screen compounds, was calculated for the two cell sources [18]. The toxicity risk of a compound, calculated as the ratio of MEC to C_max⁡_, gives an indication of the significance of the cytotoxic signals. These ratios provide an estimate of minimal safety margin [7]. Notably, both hiPSC derived hepatocytes and HepG2 had 100% sensitivity with a cutoff TR of 30.

The sensitivity in predicting various mechanisms was quite similar for hiPSC derived hepatocytes and HepG2 cells. The drug induced effects observed were comparable to those previously reported in HepG2 and primary hepatocytes (freshly isolated and cryopreserved) [9, 19]. For compounds inducing steatosis and phospholipidosis, accumulation of neutral lipids and phospholipids were observed at subcytotoxic concentrations. The simultaneous incorporation of multiple probes to the assay resulted in different mechanistic read-outs and gave a good predictivity of human toxicity.

Human iPSC derived hepatocytes failed to predict the effect on PMP when treated with tetracycline hydrochloride. However, whether this compound has an effect on PMP in intact liver is unclear. Significant changes on tetracycline induced MMP were not detected in any of the two model systems tested. Amiodarone, a cationic amphiphilic compound, infers with both mitochondrial and lysosomal function, causing steatosis and phospholipidosis [28]. HepG2 had a false negative prediction of amiodarone induced phospholipidosis. In hiPSC derived hepatocytes there was an induction of phospholipids at lower concentrations but not at higher concentrations followed by steatogenic lipid accumulation in droplets of the cells (Figures 3 and 4).

To understand the significance/relevance of HCA results, a scoring system was applied to estimate the degree of injury induced by a compound at a fixed concentration. Based on the degree of injury, the compounds were categorized as severe, moderate, mild, or nontoxic. The compounds identified with DILI potential and the order by which the compounds were categorized were in accordance with previously published data on primary human hepatocytes and HepG2 cells [7, 9, 19]. By ranking these compounds individually based on their severity, we found that the results for hiPSC derived hepatocytes were in line with FDA-approved drug labeling for the study of DILI [29]. In HepG2 cultures, the negative control sodium citrate generated a false positive score for phospholipidosis at 100 *μ*M and was thereby falsely categorized as mildly toxic. Based on the MEC and degree of injury scoring detected in this study, hiPSC derived hepatocytes are superior to HepG2 as a model for predicting phospholipidosis. Although an interesting and promising result, the present evaluation is based on only four compounds/chemicals, while a larger set of compounds with a wide range of toxicity potential has to be screened in order to validate the sensitivity and specificity of hiPSC derived hepatocytes as a predictive model system.

Presently, primary human hepatocytes (PHH) are the gold standard for liver toxicity testing and in sandwich cultures PHH can be maintained for long term up to 14 days. Chlorpromazine exposure for short and long term induces different types of hepatotoxicity in PHH sandwich cultures including steatosis revealed by transcriptomic analysis [30]. However, the interindividual variability, complications in culturing, and rapidly decreasing functionality associated with these cells have led to a wide use of immortalized cell lines [31]. HepG2 cells which are derived from a human adenocarcinoma of the liver are being preferred over HeLa, ECC-1, and CHO-K1 cell lines for hepatotoxicity studies [32, 33]. However, their biotransformation capacity via cytochrome P450 enzymes is less than 1% of normal hepatocytes [33, 34]. For many compounds, the reactive metabolite is more toxic than the parent compound [18]. To screen such compounds, improved* in vitro* model systems need to be developed. HepaRG cells, a highly differentiated hepatic cell line derived from human liver carcinoma, have been reported to have high cytochrome P450 activity, functional drug transporters, and nuclear receptors making them a promising model for bioactivation and uptake studies [35, 36]. In addition, HepaRG has been shown to be a well-suited model for studying mechanistic hepatotoxicity such as steatosis, phospholipidosis, and cholestasis [37]. Moreover, amiodarone and tetracycline induce lipid droplets in HepaRG cells as well as phospholipidosis by amiodarone [37, 38]. However, the sensitivity and predictivity of these cells in detecting hepatotoxic drugs have been shown to be much lower than cryopreserved hepatocytes [39]. Importantly, these progenitor cells originate from one individual having a specific genotype. This limitation needs also to be taken into account for assessing metabolic and toxicity studies [40]. Hepatocytes derived from hESCs and hiPSCs may bridge today's gaps in safety pharmacology and toxicology by providing more stable metabolically competent cells with a functionality comparable to freshly isolated hepatocytes [11]. The two essential bottlenecks of limited supply and batch-to-batch variability can be efficiently surpassed with hiPSC derived material. In addition, hiPSC derived hepatocytes will offer a unique possibility to design/choose a desired genetic background and phenotype of the model cells. For hiPSC derived hepatocytes to replace the existing model systems, a large compound set has to be screened and the cells must recapitulate previously established results. The possibility to correlate observed effects and pathways with an* in vivo* effect would be the most desirable form of validation. Interestingly, in our assay using hiPS derived hepatocytes, the order in which the different mechanistic effects were observed for amiodarone was similar to the clinical findings in humans reported in a case study [41].

It has been reported that conventional assays which measure late lethal events (*e.g.*, cell membrane rupture and LDH release) have poor concordance with human toxicity. A greater predictive power could be achieved when an assay can detect effects at subcytotoxic concentrations prior to the onset of general degeneration and cell death [7]. Mitochondrial potential and cellular redox states are the most important mechanisms of drug induced hepatotoxicity [9] and these two parameters had the highest sensitivity in hiPSC derived hepatocytes based on IC_50_/EC_50_ values (Table 2). The current assay using hiPSC derived hepatocytes displayed very low variance between wells, plates, and batches. The imprecision values measured for all the parameters were quite comparable between HepG2 and hiPSC derived hepatocytes and were of the same range with previous work in HepG2 cells [7]. Overall, hiPSC derived hepatocytes are as robust for live cell analysis as the well-established and very user-friendly cell line HepG2 cells (Table 5). The robustness of the hiPSC derived hepatocyte model in combination with the reported stability of important functionalities [15] also allows for long term chronic toxicity testing [16].

## 5. Conclusions

The present study shows that homogenous cultures of hiPSC derived hepatocytes can be used as a platform to assess mechanistic toxicity and reveal drug induced steatosis and phospholipidosis by image based HCA. Importantly, the hiPSC derived hepatocytes generated reproducible and consistent HCA data with low imprecision between wells, plates, and batches; thus hiPSC derived hepatocytes can serve as a robust platform for image based HCA. In addition, the hiPSC derived hepatocytes could categorize test compounds as severe, moderate, mild, or nontoxic based on the degree of injury at isomolar concentration in concordance with previously published data. Moreover, the hiPSC technology opens up further possibilities to generate infinite numbers of hepatocytes from, for example, DILI patients and individuals representing different phenotypic and genotypic variations. In addition, the development of more sensitive and complex* in vitro* toxicity models for drug screening, based on hiPSC derived hepatocytes in combination with coculturing systems with nonparenchymal liver cells or T cells, is anticipated. The present study clearly reveals the potential of the use of hiPSC derived hepatocytes in assessing hepatotoxicity* in vitro* by the use of HCA.

## Supplementary Material

The table is a scoring sheet of the compound induced changes for each HCA parameter at isomolar concentration of 100 µM (except for Amiodarone HCL at 125 µM). The scoring sheet serves to estimate the degree of injury for each compound and is adapted from Tolosa et al. (18). The order of degree of injury which the compounds are classified is compared between hiPSC derived hepatocytes (hiPS HEP) and HepG2 cell lines.

## Figures and Tables

**Table 1:** Compounds studied according to their documented mechanism of action.

Compounds	MW	CAS number	Therapeutic group/chemical class	Mechanism of toxicity
Amiodarone HCl	681.8	19774-82-4	Antiarrhythmic agent	ST, PL, MI
Doxycycline	512.94	24390-14-5	Antibiotic	MI, AP, ST
Tetracycline hydrochloride	480.9	64-75-5	Antibiotic	ST, MI

AP: apoptosis; CAS: chemical abstracts service; MI: mitochondrial impairment; MW: molecular weight; PL: phospholipidosis; ST: steatosis.

**Table 2:** IC_50_/EC_50_ values for drug induced effects on different parameters measured in hiPSC derived hepatocytes (hiPS-HEP) and HepG2.

Compound	Amiodarone		Doxycycline		Tetracycline	
Cell line	hiPS-HEP	HepG2	hiPS-HEP	HepG2	hiPS-HEP	HepG2
Fixed						
Nucleus						
Cell count	31.0	7.6	107	165	432	558
MI	95.7	*140*	47.2	*892*	**211**	*348*
Area	**2.2**	*39.5*	*96.1*	*OR*	***105***	464
Steatosis						
Count	21.0	19.5	115	260	184	***36.3***
MI	*34.1*	23.6	441	*OR*	*418*	361
Area	25.5	30.6	—	398	*213*	*287*
Phospholipidosis						
Count	6.1	8.9	NA	NA	NA	NA
MI	6.0	*35.6*	NA	NA	NA	NA
Area	***4.5***	7.3	NA	NA	NA	NA

Live						
Nucleus						
Cell count	21.9	**7.2**	69.4	**120.3**	243	836
MI	*22.4*	25.0	*120*	153	*380*	959
Area	10.4	*29.1*	*204.3*	—	211	336
MMP						
MI	20.8	*345*	644	622	—	***265***
ROS						
MI	26.5	***4.6***	***10.5***	***115***	160	1149
PMP						
MI	*42.2*	*147*	**26.9**	*OR*	*OR*	*OR*

Four-parameter variable slope curve fit using the least squares method was applied for generating dose response curves in GraphPad Prism software. Parameters with the lowest and second lowest IC_50_/EC_50_ values are denoted in bold; values in italics had an ambiguous near complete dose response curve fit; [OR], curves which were out of range. Values denoted are mean values from three different experimental batches (*N* = 3). All values are represented as *μ*M.

**Table 3:** Cytotoxic effects of the tested compounds in hiPSC derived hepatocytes (hiPS-HEP) and HepG2: minimal effective concentration and toxicity risk steatotic risk index.

Cell system	Compound	Fixed assay									Live assay						*C*_max⁡_^a^	TR^b^	SRI^c^	
		V	NC		S			PL			V	NC		MMP	ROS	PMP			Lipid	ROS
		CC	MI	A	C	MI	A	C	MI	A	CC	MI	A	MI	MI	MI				
hiPS-HEP	AMD	39.5	39.5	—	39.5	125	39.5	**12.5**	**12.5**	**12.5**	39.5	39.5	—	39.5	39.5	125	2.2	5.7	18	18
	DOX	100	—	316	316	1000	—	—	—	—	1000	100	100	1000	—	**31.6**	8.8	3.6	36	>114
	TET	1000	—	**316**	**316**	1000	—	—	—	—	1000	—	**316**	—	**316**	—	14.2	22.3	22	22
	SC	—	—	—	—	—	—	—	—	—	—	—	—	—	—	—	NA	NA	NA	NA

HepG2	AMD	**4**	39.5	125	39.5	39.5	125	—	—	—	12.5	39.5	39.5	12.5	12.5	125	2.2	1.8	18	6
	DOX	**100**	1000	1000	316	1000	—	—	—	—	316	—	—	1000	316	1000	8.8	11.4	36	36
	TET	316	**100**	316	**100**	**100**	—	—	**100**	—	—	1000	316	—	1000	1000	14.2	7.0	7	70
	SC	—	—	—	—	—	—	—	—	—	—	—	—	—	—	—	NA	NA	NA	NA

Mechanism affected at the lowest concentration is denoted in bold. Statistical significance was performed using one-way ANOVA followed by Dunnett's test. AMD: amiodarone HCl; DOX: doxycycline; TET: tetracycline; V: viability; NC: nuclear changes; S: steatosis; PL: phospholipidosis; MMP: mitochondrial membrane potential; ROS: reactive oxygen species; PMP: plasma membrane permeability; CC: cell count; MI: mean intensity; A: area; C: droplet count. ^a^Maximum plasma concentration of the drug (*C*_max⁡_), ^b^The toxicity risk (TR) for each compound was defined as ratio of minimal effective concentration to the maximum plasma concentration of the drug (*C*_max⁡_). ^c^Steatotic risk index (SRI) was calculated as the ratio of the MEC for neutral lipid accumulation or for ROS generation to the *C*_max⁡_.

**Table 4:** Categorizing compounds based on their degree of injury at isomolar concentration of 100 *µ*M for each parameter measured.

Degree of injury	hiPSC derived hepatocytes	HepG2
Severe hepatotoxicity	Amiodarone HCl^*∗*^	Amiodarone HCl^*∗*^
Moderate hepatotoxicity	Doxycycline	
Mild hepatotoxicity	Tetracycline HCl	Doxycycline, tetracycline HCl, sodium citrate
Nontoxic	Sodium citrate	

Refer supplementary-I Table 1 for the scoring sheet on degree of injury calculation (see Supplementary Material available online at http://dx.doi.org/10.1155/2016/2475631).  ^*∗*^Degree of injury measured at 125 *µ*M for amiodarone.

**Table 5:** Comparison of assay imprecision for each parameter measured in hiPSC derived hepatocytes (hiPS-HEP) and HepG2.

Assay parameter	Variation between wells in a plate		Variation between plates in a batch		Variation between batches	
	hiPS-HEP	HepG2	hiPS-HEP	HepG2	hiPS-HEP	HepG2
	CV% ± SD	CV% ± SD	CV%	CV%	CV%	CV%
CC (fixed)	6.8 ± 4.8	8.9 ± 2.6	13.0	4.1	26.7	22.6
CC (live)	15.3 ± 12.3	18.9 ± 17.3	25.8	25.5	35.3	35.0
NC						
MI	2.4 ± 1.3	1.0 ± 0.6	6.0	22.3	4.1	12.9
Area	2.3 ± 1.1	2.2 ± 0.9	6.4	6.7	5.5	6.9
ST						
MI	6.3 ± 2.7	5.0 ± 4.6	9.9	5.7	21.4	8.6
Area	3.9 ± 3.1	3.5 ± 4.5	1.1	10.7	6.6	9.5
PH						
MI	3.2 ± 1.5	1.8 ± 1.4	7.9	4.5	8.1	4.0
Area	2.4 ± 1.0	10.2 ± 10.5	18.1	37.0	13.3	2.8
MMP						
MI	4.7 ± 6.2	3.4 ± 2.7	12.3	22.1	6.8	22.4
ROS						
MI	0.9 ± 0.84	4.1 ± 2.8	4.0	7.3	5.8	5.2
PMP						
MI	4.8 ± 3.02	2.6 ± 2.0	3.9	14.7	0.6	23.0

Imprecisions of cell-to-cell within a well, well-to-well within a plate, plate-to-plate within a batch, and batch-to-batch are compared for all parameters measured. For calculating well-to-well variance, a mean, SD, and CV% were calculated for every control well (*N* = 3) and an average CV% of all the eight plates was reported for well-to-well variation. The well-to-well mean values for each parameter within a batch (*N* = 2) were then averaged and its CV% was calculated. The average CV% from the four batches was used to show plate-to-plate variation. For batch-to-batch variance, the mean values from each plate were averaged and its CV% was reported (*N* = 4).
