# Supplementary material for: High Content Analysis of Human Pluripotent Stem Cell Derived Hepatocytes Reveals Drug Induced Steatosis and Phospholipidosis
Source: Stem Cells Int. 2016 Jan 5;2016:2475631. doi: 10.1155/2016/2475631 (PMC4736406; doi:10.1155/2016/2475631)
Supplement: Supplementary file 1 — The table is a scoring sheet of the compound induced changes for each HCA parameter at isomolar concentration of 100 µM (except for Amiodarone HCL at 125 µM). The scoring sheet serves to estimate the degree of injury for each compound and is adapted from Tolosa et al. (18). The order of degree of injury which the compounds are classified is compared between hiPSC derived hepatocytes (hiPS HEP) and HepG2 cell lines. [file 2475631.f1.pdf]

Supplementary – I

Table 1: Scoring sheet to estimate the degree of injury

Compounds	Model system	FIXED									LIVE							Severity
		Nucleus			Neutral Lipids			Phospholipids			Nucleus			MMP	ROS	PMP		
		CC	MI	Area	C	MI	Area	C	MI	Area	CC	MI	Area	MI	MI	MI		
Amiodarone HCL	hiPS HEP	3	0	0	3	1	0	3	0	1	3	3	0	0	3	0		20
	HepG2	3	1	1	2	3	0	0	0	1	3	2	1	1	1	1		20
Doxycycline	hiPS HEP	1	0	0	2	1	0	0	0	0	1	0	2	0	0	1		8
	HepG2	1	0	0	1	0	0	1	0	0	0	0	0	0	0	0		3
Tetracycline HCL	hiPS HEP	0	0	0	2	0	0	1	0	0	0	0	0	0	0	0		4
	HepG2	0	0	0	2	1	0	0	0	0	0	0	0	0	0	0		4
Sodium Citrate	hiPS HEP	0	0	0	0	0	0	0	0	0	0	0	0	0	0	0		0
	HepG2	0	0	0	0	0	0	1	0	0	0	0	0	0	0	0		1

In order to classify compounds according to its toxicity potential, the level of change was calculated for each parameter at isomolar concentration of 100 µM (except for Amiodarone HCL at 125 µM). The scoring sheet used to estimate the degree of injury was adapted from Tolosa et al. (18). Four different scores were assigned according to the level of variation when compared to the vehicle control; 0 (variation lower than 20%), 1 (variation ± 20-40%), 2 (variation ± 40-60%), and 3 (variation ± 60-100%). The percentage change for steatosis and phospholipidosis were in a different range, so different levels were established. The scores were assigned as; 0 (variation lower than 25%), 1 (variation 25-150%), 2 (variation 150-300%), and 3 (variation >300%). The individual scores for each parameter were summed up to estimate the degree of injury of the compound. The compounds were classified based on the degree of

injury as severely toxic ($\geq$15), moderately toxic (6-15), mildly toxic (1-5) and non-toxic. The order in which the compounds were classified was compared between hiPSC derived hepatocytes (hiPS HEP) and HepG2 cell lines.
